# Prediction of road dust concentration in open-pit coal mines based on multivariate mixed model

**DOI:** 10.1371/journal.pone.0284815

**Published:** 2023-04-26

**Authors:** Meng Wang, Zongwei Yang, Caiwang Tai, Fan Zhang, Qiaofeng Zhang, Kejun Shen, Chengbin Guo

**Affiliations:** 1 College of Mining, Liaoning Technical University, Fuxin, Liaoning, China; 2 School of Civil Engineering, Wuhan Univerisity, Wuhan, Hubei, China; 3 Xima Coal Mine of Shenyang Coking Coal Co. Ltd, Shenyang, Liaoning, China; 4 Mixlinker Networks (Shenzhen) Inc, Shenzhen, Guangzhou, China; University of Wisconsin-Eau Claire, UNITED STATES

## Abstract

The problem of dust pollution in the open-pit coal mine significantly impacts the health of staff, the regular operation of mining work, and the surrounding environment. At the same time, the open-pit road is the largest dust source. Therefore, it analyzes the influencing factors of road dust concentration in the open-pit coal mine. It is of practical significance to establish a prediction model for scientific and effective prediction of road dust concentration in the open pit coal mine. The prediction model helps reduce dust hazards. This paper uses the hourly air quality and meteorological data of an open-pit coal mine in Tongliao City, Inner Mongolia Autonomous Region, from January 1, 2020, to December 31, 2021. Create a CNN-BiLSTM-Attention multivariate hybrid model consisting of a Convolutional Neural Network (CNN), a bidirectional long short-term memory neural network (BiLSTM), and an attention mechanism, Prediction of PM2.5 concentration in the next 24h. Establish prediction models of parallel and serial structures, and carry out many experiments according to the change period of the data to determine the optimal configuration and the input and output size. Then, a comparison of the proposed model and Lasso regression, SVR, XGBoost, LSTM, BiLSTM, CNN-LSTM, and CNN-BiLSTM models for short-term prediction (24h) and long-term prediction (48h, 72h, 96h, and 120h). The results show that the CNN-BiLSTM-Attention multivariate mixed model proposed in this paper has the best prediction performance. The mean absolute error (MAE), root mean square error (RMSE), and coefficient of determination (R^2^) of the short-term forecast (24h) are 6.957, 8.985, and 0.914, respectively. Evaluation indicators of long-term forecasts (48h, 72h, 96h, and 120h) are also superior to contrast models. Finally, we used field-measured data to verify, and the obtained evaluation indexes MAE, RMSE, and R^2^ are 3.127, 3.989, and 0.951, respectively. The model-fitting effect was good.

## 1. Introduction

China ’s ’ coal industry policy ’ and ’ coal mine safety production ’ 14th Five-Year Plan ’ clearly stated that open-pit coal mines should prioritize development [1]. With the increase of opencast coal mining, the dust problem is becoming more serious. The dust not only causes pollution to the surrounding environment but also causes great harm to the physical and mental health of the human body and affects the working efficiency and safety production of the mining area. And in the mining operation of the open-pit coal mine, the dust of transportation roads is the largest source of dust, accounting for about 70% -90% of the total mineral dust [2]. According to the International Organization for Standardization (ISO), dust is a suspended solid with a particle size of fewer than 75 μm, and particles with a particle size of less than 10μm are called floating dust, including PM2.5 and PM10. PM2.5 is particles with a particle size of less than 2.5μm in the air, PM10 is particles with a particle size of less than 10μm in the air, and particles with a particle size of more than 10μm are called dust fall. Dustfall can settle to the ground in a short time under the action of gravity. At the same time, PM2.5 and PM10 can be suspended in the air for a long time due to their small size and vital activity, and PM2.5 is more likely to carry some toxic and harmful substances, which is more damaging to human health and the surrounding environment [3]. Therefore, it is necessary to study the prediction of road dust concentration in the open-pit coal mine for everyday mining work and workers’ health. In terms of PM2.5 concentration prediction, there are three main methods: prediction method based on a physical simulation model, prediction method based on a statistical model, and prediction method based on machine learning.

(1) The prediction method based on the physical simulation model is to simulate the diffusion process and environmental factors of pollutant particles in the air by computer simulation to achieve the purpose of prediction. Chemel C et al. used chemical transport models to predict future changes in PM2.5 concentrations in the UK in combination with pollution emissions and meteorological conditions [4]. Y Hu et al. used the Gaussian plume diffusion model and combined multiple linear regression to predict PM2.5 concentrations in Xi ’a China from meteorological factors and pollution source diffusion [5]. G Zhou et al. established a pollutant prediction system RAEMS for eastern China to predict PM2.5 concentration by simulating pollutant source emissions, atmospheric physics, atmospheric chemical processes, and regional traffic volume [6]. J Pan et al. integrated three prediction systems (CMAQ, CAMx, and NAQPMS) to predict the concentration of PM2.5 in Beijing and systematically considered the impact of the weather system and medium-scale and small-scale meteorology on the transport, diffusion, and transformation of pollutants [7]. Thongthammachart T et al. combined random forest with WRF/CMAQ model to predict PM2.5 concentration in Kansai, Japan [8]. Cheng X et al. developed a 3DVR assimilation system based on the coupling model of CRTM and WRF-Chem to predict the 24-hour PM2.5 concentration in Beijing [9]. Although the prediction effect of the simulation model is better, building a physical simulation model needs to combine many complex factors such as topography, pollution sources, pollutants, and meteorology. It requires high data detail, constantly updating pollution source information, and information acquisition is challenging, excessive consumption of resource costs and labor costs, implementation is complex, and this method has certain defects.

(2) The prediction method based on the statistical model mainly analyzes historical data and uses specific mathematical methods for scientific processing, revealing the stable relationship between relevant variables, obtaining the change law of dust concentration, and predicting future changes. Statistical model prediction methods include multiple linear regression prediction, grey theory, Markov model, exponential smoothing, autoregressive moving average model, and so on. Elbayoumi et al. used outdoor PM10, PM2.5, CO, CO_2_ concentration, wind speed, air pressure, and relative humidity as input to establish a multiple linear regression model of PM2.5 and PM10 to predict indoor PM2.5 and PM10 concentration. Because the atmospheric process is nonlinear, the linear regression method is difficult to accurately describe the nonlinear relationship, resulting in the prediction effect could be wrong [10]. X Xi et al. built a combination model of grey system prediction model GM (1,1) and ARMA (p, q) to predict the concentration of PM2.5 in Xi’an. The research results show that the relative error of the combination model is the smallest [11]. Y Zhang et al. screened out the main influence parameters on the prediction of PM2.5 concentration through the MIV algorithm. They built the ARIMA model to predict the concentration of PM2.5 in Guilin. The results showed that the MIV screening parameters could improve the prediction accuracy of the model [12]. X Zhang et al. used an exponential smoothing algorithm to predict PM2.5 concentrations and used a Markov model to correct them. The prediction results were more accurate than uncorrected prediction models [13]. Y Ju et al. compared and analyzed the predictive ability of the multiple linear and nonlinear regression models on PM2.5 concentration in Tianmen City. The results showed that the numerous nonlinear regression model had an excellent predictive effect [14].

(3) The prediction method based on machine learning uses the learning algorithm to analyze a large number of related historical data and learn the nonlinear transformation law between data to achieve the purpose of prediction. It is a prediction method commonly used by scholars in recent years. The widely used machine learning models are random forest, support vector machine, artificial neural network, etc. Compared with traditional prediction methods, the prediction method based on machine learning simplifies the prediction process and has advantages in prediction accuracy. Y Wang established a random forest-Markov model to predict the dust concentration in Haerwusu open-pit mine. She constructs a hybrid model based on the random forest and Markov models. The results show that the hybrid model has a better prediction effect than the single random forest model [15]. R Wang et al. combined a support vector machine with a genetic algorithm, trained and tested the model using common data sets, and compared it with other machine learning methods. The results show that GA-XVR has a higher prediction accuracy [16]. J Yin et al. established a multi-level residual modified least squares support vector regression model based on wavelet decomposition to predict PM2.5 concentration in Jinan, China. The results show that the model predicts better, especially for heavy-pollution weather [17]. W Zhong et al. first used the mRMR algorithm to select data features and then used the XGBoost model to predict the concentration of PM2.5 in the next hour. The results showed that selecting features first can improve prediction accuracy [18]. J Zhong et al. built a LightGBM algorithm based on meteorological data to predict hourly PM2.5 concentration [19]. J Kang et al. built KNN, BP, SVR, GPR, XGBoost, and random forest models to predict PM2.5 concentration. The comparative experimental results show that the prediction accuracy of the XGBoost model is higher than other models [20].

As a new research direction in machine learning, deep learning has gradually become a prediction method with frequent use and better prediction effect. Deep learning enhances the representation ability of complex features. The research on PM2.5 concentration prediction using deep learning algorithms mainly applies a deep neural network model to extract sufficient historical information in time series to predict PM2.5 concentration. Standard deep neural network models include Recurrent Neural Networks (RNN), Convolutional Neural Networks (CNN), and Deep Belief Networks (DBN). Y Zhang predicted the dust concentration of the Haerwusu open-pit mine using the LSTM recurrent neural network model and compared it with the traditional regression and random forest models. The results show that the prediction results of the LSTM recurrent neural network model are better than the other two models [21]. M Teng et al. proposed a hybrid model combining long short-term memory neural network and convolutional neural network combined with the monitoring information of adjacent stations to predict PM2.5 concentration, and compared with the single model. The results show that the hybrid model has higher prediction accuracy [22]. F Jiang et al. used a hybrid model of CEEMDAN and deep-time convolutional neural networks to predict hourly PM2.5 concentrations. The prediction results show that the hybrid model can achieve better prediction accuracy than a single model [23]. J Kang et al. improved XGBoost and LSTM to obtain a variable weight combination model XGBoost-LSTM (Variable) to predict the hourly concentration of PM2.5 in Shanghai. The processed data sets were input into two models for prediction, and then the final prediction results were obtained using the variable weight combination method based on residual improvement. They chose XGBoost, LSTM, and SVR for comparative experiments. The results show that the prediction accuracy of the hybrid model is better than that of the single model [24]. M Zhu and J Xie established a CNN-BiLSTM mixed model to predict PM2.5 concentration. The experimental results show that combining the monitoring data of the target site and nearby sites can improve prediction accuracy [25]. Kow P Y et al. proposed a fusion of multiple convolutional neural networks (MCNN) and back-propagation neural network (BPNN) and used MCNN-BP to predict PM2.5 at 74 stations in Taiwan [26]. K Gu et al. proposed a composite LSTM model GRA-ICEEMD-LSTM. They use GRA for variable screening, ICEEMD for data processing, and LSTM to predict PM2.5 concentration. The results show that the composite LSTM model has higher accuracy and shorter training time than the single prediction model [27]. Kim Yong-been et al. compared the PM2.5 prediction performance of LSTM, GRU, and BiLSTM models using the data from Seoul, Daejeon, and Busan in South Korea. The results showed that BiLSTM has high prediction accuracy in long-term prediction [28]. Scholars ’ research has developed from a single neural network model to a hybrid model. Facts have proved that the hybrid model’s prediction accuracy is better than a single model. There are many influencing factors for dust concentration, but each aspect affects dust concentration changes differently. Currently, most prediction methods based on CNN and LSTM do not consider this. Therefore, by introducing the attention mechanism, the prediction model can obtain the influence of variable characteristics at different times in the past on the future dust concentration.

Therefore, this paper proposes a CNN-BiLSTM-Attention hybrid model based on the Keras framework to predict road dust concentration in open-pit coal mines. Keras can easily define and train almost all types of deep learning models [29]. CNN can extract sufficient feature information from input variables [30]. BiLSTM as a variant of LSTM, can not only solve the problem of gradient disappearance or gradient explosion of a recurrent neural network but also obtain data information from the forward (from left to right) and reverse (from right to left) of the data. At the same time, it effectively uses the characteristics of the future and the past [31]. At the same time, the model introduces an attention mechanism to analyze the importance of all features and gives corresponding weights. The model obtains the proportion of the influence of different characteristics on dust concentration and outputs the final forecast results [32]. The hybrid model combines the aspects and advantages of each algorithm to improve the prediction accuracy and better meet the needs of actual prediction. The address of the source code of the prediction model on GitHub is https://github.com/Zongwei-Yang/Thesis-code.git.

The content of this paper is as follows. The first part is the introduction. The second part is the basic theory of each model. The third part is the related processing and analysis of experimental data. The fourth part is the construction of the prediction model. The fifth part is the testing process. The sixth part is the conclusion.

## 2. Basic theory of model

### 2.1 CNN

**Fig 1:**
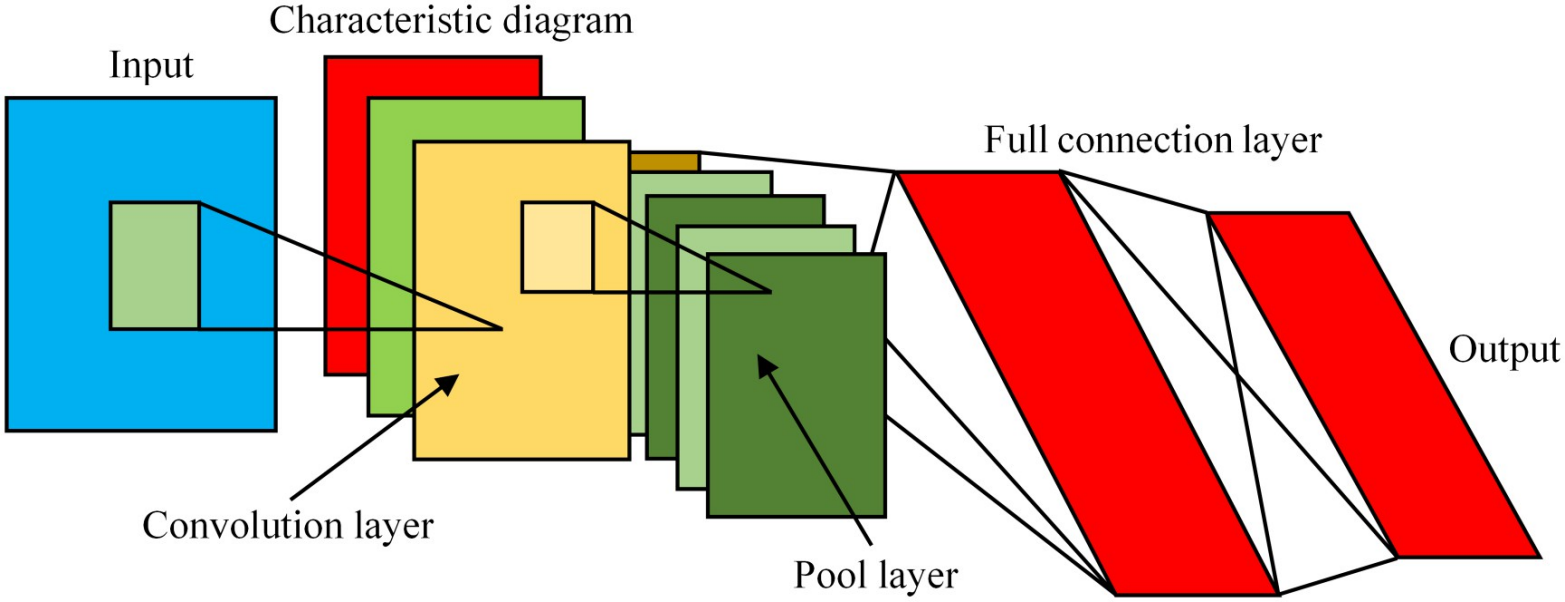
Basic structure of CNN model.

Convolutional Neural Network (CNN) is a feed-forward neural network with convolution computation and deep structure. The structure of the feed-forward neural network adopts a one-way and multi-layer mode. The whole neural network transmits signals in one direction, and neurons are arranged in layers. At the same time, each layer contains several neurons. Each layer of neurons is only connected to the neurons of the previous layer, receives the neurons of the last layer, and then outputs to the next layer. The overall structure of the CNN model consists of five parts: input layer, convolution layer, pooling layer, whole connection layer, and output layer. The input layer of the convolution neural network can process multi-dimensional data. The function of the convolution layer is to extract the feature of input data and then reduce the dimension of the feature extracted by the convolution layer through the pooling layer to further extract the feature. Finally, the training results of the model are output through the complete connection layer and the output layer [33, 34]. Fig 1 shows the basic structure of the CNN model.

The mathematical expression of CNN model convolution process is:

$$Z^{l+1}(i,j)=\left[Z^{l}⊗w^{l+1}\right](i,j)+b=\sum_{k=1}^{K_{l}}\sum_{x=1}^{f}\sum_{y=1}^{f}\left[Z_{k}^{l}\left(s_{0}i+x,s_{0}j+y\right)w_{k}^{l+1}(x,y)\right]+b$$ (1)

$$(i,j)\in \left\{0,1,\ldots L_{l+1}\right\}L_{l+1}=\frac{L_{l}+2p-f}{s_{0}}+1$$ (2)

In the form, *Z*^*l*^ is the convolution input of the layer *l+1*; *Z*^*l+1*^ is the convolution output of the layer *l+1*; *L*_*l+1*_is the size of *Z*_*l+1*_; *Z(i*,*j)* is the pixel of the corresponding feature graph; *K* is the number of characteristic graph channels; *f* is the convolution kernel size; *S*_*o*_ is convolution step length; *p* is zero fill layers; *w* is the weight coefficient; *b* is the deviation. The sum part in the formula is equivalent to solving a cross-correlation.

The activation function is ReLU. Compared with the Sigmoid and Tanh functions, the ReLU function can improve convergence speed [35]. Because of its simple gradient solution formula, it can effectively improve the problem of gradient disappearance and gradient explosion [36]. The following is the mathematical expression of the ReLU function.


$$ReLU(x)=\left\{\begin{array}{c} x,x\geq 0\\ 0,x<0 \end{array}\right.$$ (3)

The data needed in this paper are time series data. Since the time series data only change along the time axis, it is more effective to use a one-dimensional convolution neural network (1D-CNN) to process [37]. Therefore, this paper uses the 1D-CNN model to extract the local characteristics of data.

### 2.2 BiLSTM

The recurrent neural network (RNN) has a specific advantage in learning the nonlinear characteristics of the sequence, and it has a good effect in processing time series data. However, the recurrent neural network has a long-term dependence problem. When the input time series data is too long, the model will lose the ability to learn early data. To solve this problem, scientists proposed the variant structure of the recurrent neural network, the long-term and short-term memory network (LSTM), based on the ‘gate’ structure [38].

**Fig 2:**
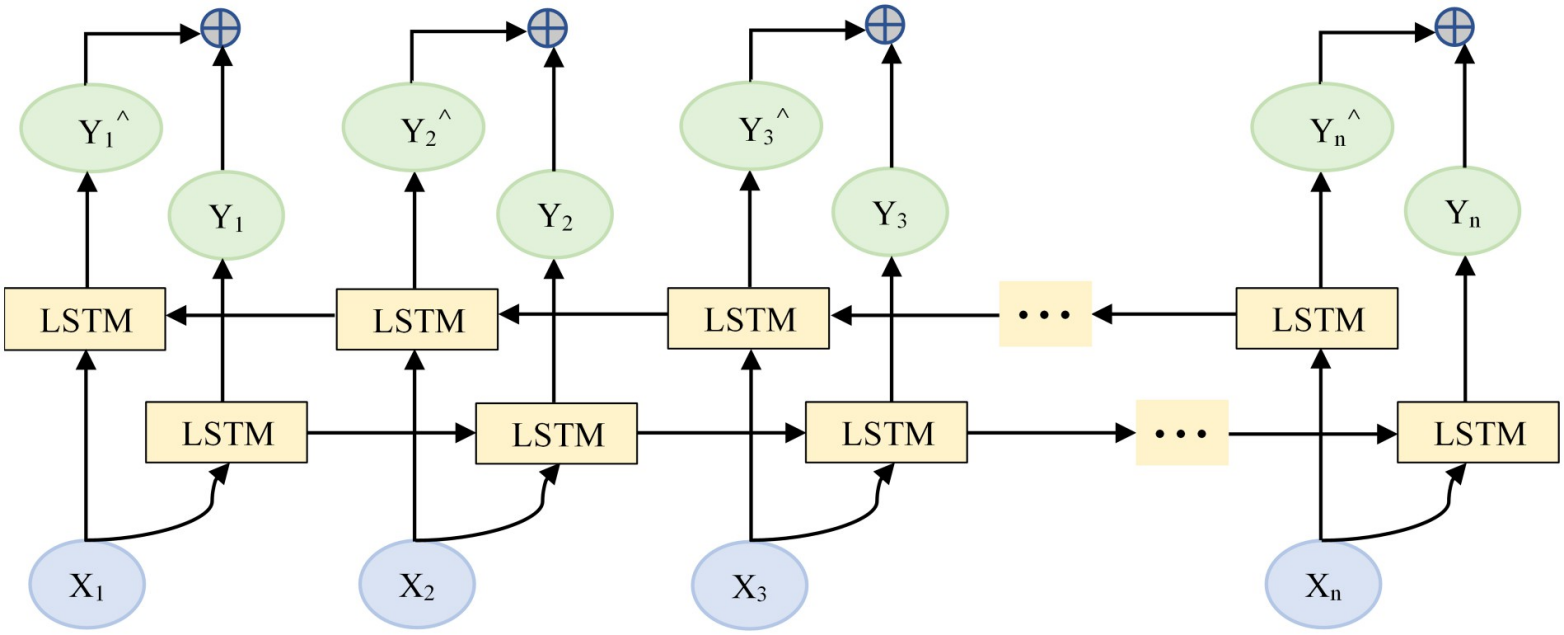
BiLSTM model basic structure.

The LSTM model effectively solves the problems of gradient disappearance and gradient explosion in the recurrent neural network [39]. Still, in model training, the LSTM model continuously learns in the time order from front to back, so it can only know certain sample data information before a moment. Unable to consider the data information after this moment. This training method leads to a low data utilization rate and cannot fully mine the inherent characteristics of the data. To solve this problem, the forward LSTM can add a reverse LSTM to form a bidirectional extended short-term memory network (BiLSTM) for prediction. The BiLSTM model comprises a forward LSTM layer and a reverse LSTM layer. During the training process, the internal relationship between the data at a specific moment, historical data, and future data to obtain the global data characteristics [40]. Improve the utilization rate of sample data and prediction accuracy rate to a certain extent. Fig 2 shows the basic structure of the BiLSTM model.

The calculation process is as follows:

$$h_{t}^{F}=f^{F}\left(w_{1}x_{t}+w_{2}h_{t-1}^{F}\right)$$ (4)

$$h_{t}^{R}=f^{R}\left(w_{3}x_{t}+w_{5}h_{t+1}^{R}\right)$$ (5)

$$h_{t}=f\left(w_{4}h_{t}^{F}+w_{6}h_{t}^{R}\right)$$ (6)

In the formula, *x*_*t*_ is the current input; *hF t-1*is the output state of the forward LSTM layer at the previous moment; *hR t+1*is the output state of the inverse LSTM layer at the previous moment; *w*_*1*_, *w*_*3*_ are the weight matrix from the input layer to the forward and reverse LSTM layers; *w*_*2*_, *w*_*5*_ is the weight matrix from forward and reverse LSTM layer to its own transfer layer; *w*_*4*_, *w*_*6*_ are the weight matrix from the forward and reverse LSTM layers to the output layer; *f* ^*F*^*(•)*, *f* ^*R*^*(•)* are the cell computing process; *f(•)* is the splicing function of the output result of the forward and reverse LSTM layers; *hF t*is the output result of the forward LSTM layer; *hR t*is the output of the reverse LSTM layer; *h*_*t*_ is the final output.

### 2.3 Attention mechanism

**Fig 3:**
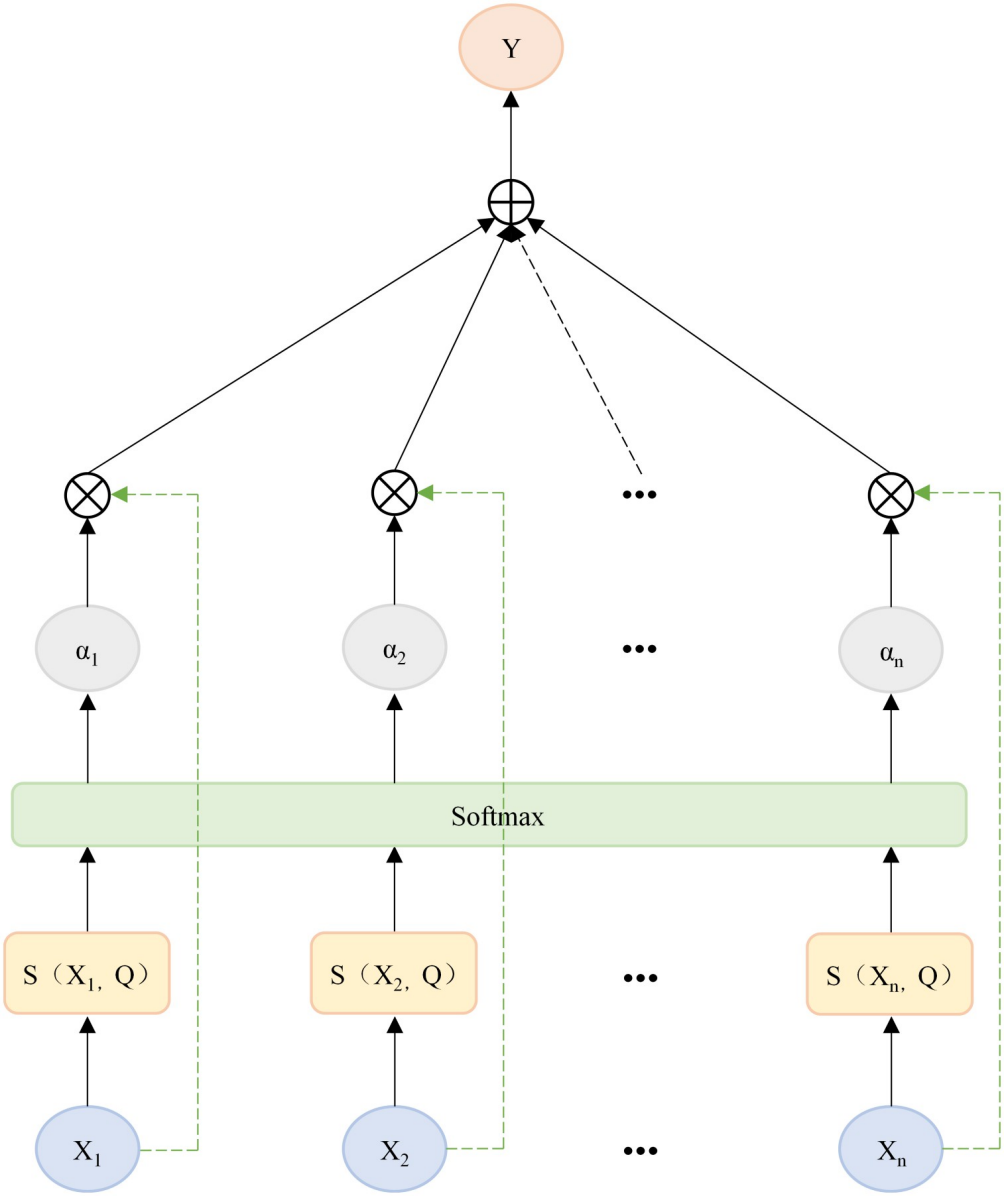
Attention mechanism structure diagram.

When processing long sequence multidimensional feature data, different variable characteristics affect the target variable differently. Important features contain more key information and impact the target variable, so by introducing an attention mechanism to give each variable feature different weights and highlight key information, it is possible to mine data association features more effectively. At the same time, it can solve the problem of information overload, significantly reduces model computation, and make reasonable use of computing resources to achieve the best model training effect [41], Fig 3 shows the structure of the attention mechanism.

First, calculate the similarity score of PM2.5 concentration and other variables. Then, use the Softmax function to normalize the obtained similarity scores and get the attention distribution for each variable. Finally, the input variables other than the target variable and the attention distribution are weighted and summed to obtain the final attention value. The calculation process is as follows:

$$S\left(X_{n},Q\right)=v^{T}\text{tanh}\left(WX_{n}+UQ\right)$$ (7)

$$\alpha_{n}=Softmax\left(S\left(X_{n},Q\right)\right)=\frac{\text{exp}\left(S\left(X_{n},Q\right)\right)}{\sum_{j=1}^{N}\text{exp}\left(S\left(X_{j},Q\right)\right)}$$ (8)

$$A_{n}=Att(X,Q)=\sum_{n=1}^{N}\alpha_{n}X_{n}$$ (9)

In the formula, *v*^*T*^ is the weight vector, *W*, *U* is the weight matrix, *Q* is the target variable, *X*_*n*_ is an input variable other than the target variable, *α*_*n*_ is the attention distribution, *A*_*n*_ is the final attention value.

## 3. Data processing analysis

### 3.1 Research objective

The research object of this paper is road dust in opencast coal mines. International Organization for Standardization (ISO) regulations, dust is suspended solids with particle size less than 75μm, including PM2.5 and PM10. During the mining process of open-pit coal mines, various techniques can generate dust. However, according to related research, the dust production of the open-pit coal mine transportation road has reached 70%-90% of the total dust production of the whole mine. In order to control dust pollution in the entire open-pit coal mine, it should start with the transportation road of the open-pit coal mine. The main influencing factors of road dust concentration in open-pit coal mines are geographical, environmental, human, and natural. The geographical environment factors mainly include the location of the open-pit coal mine, the open-pit coal mine road area, and the road structure. Human factors mainly include fugitive dust caused by moving transport vehicles and artificial watering and dust reduction measures; Natural factors include pollutants in the air and meteorological conditions. However, geographical and environmental factors generally do not change much and can be regarded as fixed factors. Human factors are uncertain, and relevant influencing factors cannot form a complete time series; therefore, the dust concentration change directly reflects these two factors’ influence on dust concentration. Studies have shown that dust accumulation in open-pit coal mines is mainly affected by temperature inversion. Due to the unique topographic and geomorphological characteristics of open-pit coal mines, there is often an inversion layer in the upper part of open-pit coal mines. Dust cannot spread outside the pit, causing dust concentration to rise. Wind speed, temperature, and ozone will affect the formation of the inversion layer, thus affecting the concentration of dust. Atmospheric gaseous pollutants (sulfur and nitrogen oxides) can through various chemical and physical processes form PM2.5. Natural factors are constantly changing. This change affects the change of dust concentration at any time. Therefore, this paper mainly studies the prediction of road dust concentration in opencast coal mines from natural factors.

### 3.2 Research data

**Fig 4:**
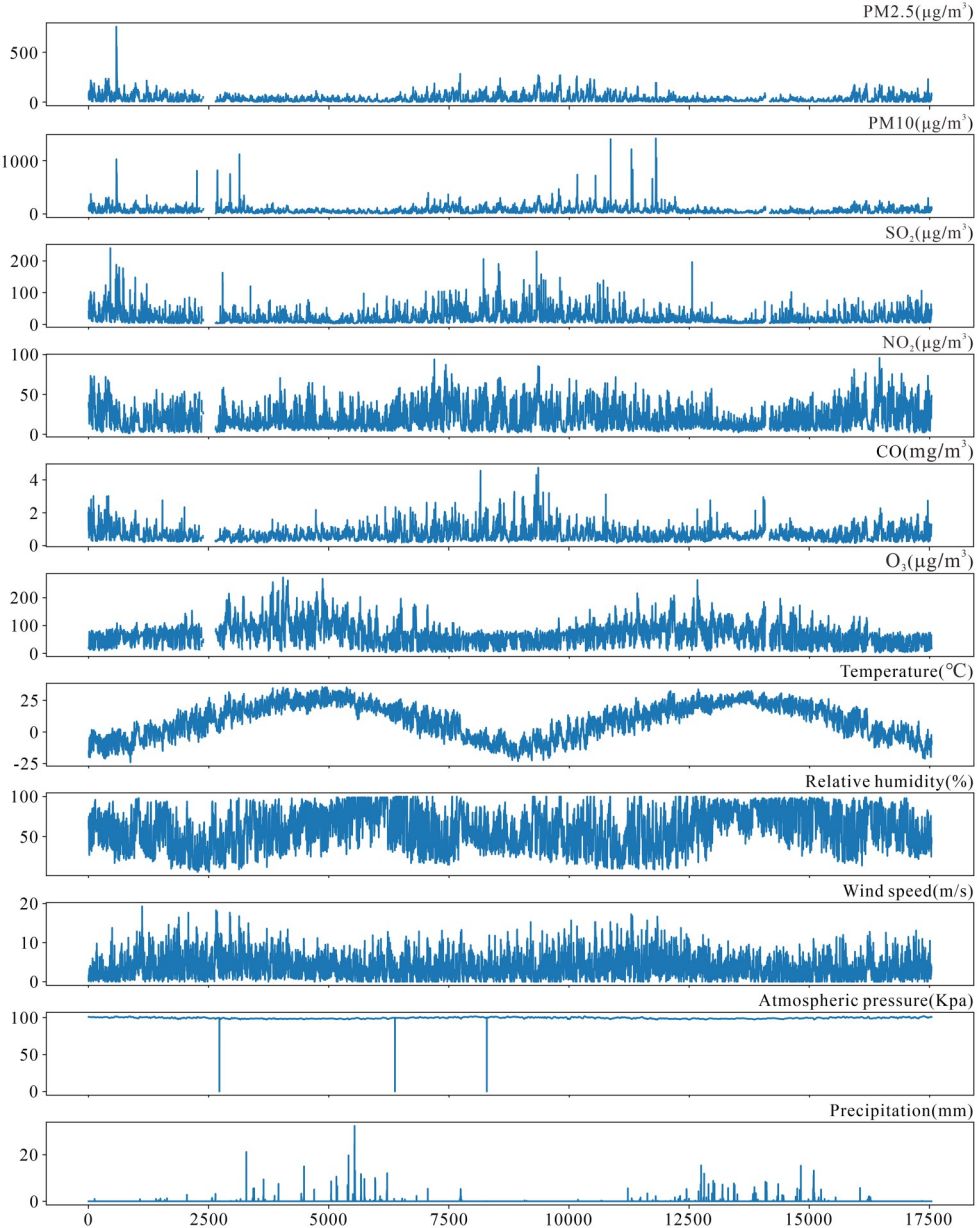
Raw data time series.

Since training deep learning models requires a large amount of data, the original data’s unit time span determines the prediction cycle’s length. To achieve short-term and long-term forecasts, the data set for training the model in this paper is the hourly air quality data and meteorological data from January 1, 2020, to December 31, 2021, an open-pit coal mine in Tongliao City, Inner Mongolia Autonomous Region. A total of 17520 sets of data includes 12 types of factors, respectively PM2.5, PM10, SO_2_, NO_2_, CO, O_3_, temperature, relative humidity, wind speed, wind direction, precipitation, and atmospheric pressure. After model training is complete, use the measured data of dust monitoring equipment to test the model’s performance. Table 1 shows the variables included in the dataset, Fig 4 offers each variable’s original data time series.

**Table 1:** Variables included in the dataset.

Air quality data	Unit	Meteorological data	Unit
PM2.5	μg**/**m^3^	Temperature	℃
PM10	μg**/**m^3^	Relative humidity	%RH
SO_2_	μg**/**m^3^	Wind speed	m**/**s
NO_2_	μg**/**m^3^	Wind direction	-
CO	mg**/**m^3^	Precipitation	mm
O_3_	μg**/**m^3^	Atmospheric pressure	KPa

As shown in the figure above, the horizontal axis represents 17575 sets of data in hours from January 1, 2020, to December 31, 2021. The vertical axis represents the value of each variable. As can be seen from the figure, the temperature value shows a pronounced periodic change. The concentrations of SO_2_, NO_2_, CO, and O_3_, relative humidity, and wind speed also have certain periodic changes. Therefore, determine the size of the input variable of the prediction model by the length of the period change. To a certain extent, we can improve the model’s prediction accuracy. But from the above figure, it is evident that there are missing values and outliers in the dataset, so these data need to preprocess to enhance the quality of sample data. It is a prerequisite for further analysis of the data.

### 3.3 Data processing

#### 3.3.1 Data preprocessing

(1) Missing values and outliers

During data collection, uncontrollable factors such as sensor failure, network problems, and external bad weather maybe cause missing and abnormal data. Missing and abnormal values affect further data analysis and model training. First, fill the missing values in the dataset with NaNs via Python, then use the mean method to fill in the missing values. For the processing of outliers, we can use the quartile analysis method to detect outliers. Let the first, second, and third quartiles are by Q_1_, Q_2_, and Q_3_, respectively. Q_1_, Q_2_, and Q_3_ are the values at the 25%, 50%, and 75% positions after all the values in the sample data are arranged from small to large. The values less than Q_1_-1.5(Q_3_- Q_1_) or greater than Q_3_+1.5(Q_3_- Q_1_) are outliers. Since the sample data is time series data, we could use the mean value of the data before and after the outliers to correct it.

(2) Data normalization

This paper studies a multivariate problem. The essence of the training model is to establish the relationship between different variables, but different variables have different physical meanings and dimensions. If it is not processed, it will seriously affect the training effect of the model, so all variables need to be normalized and scale all variable data between 0 and 1 by a certain ratio. Processed by normalization, it can speed up the convergence of the model, reduce training time, and improve the model’s prediction accuracy. The calculation formula for data normalization is as follows:

$$x^{*}=\frac{x-\text{min}x}{\text{max}x-\text{min}x}$$ (10)

In the formula, x* is the normalized result, *x* is the original data, min *x* is the smallest value in the data, and max *x* is the maximum value in the data.

#### 3.3.2 Data set partitioning

The multivariate mixed model established in this paper belongs to the supervised learning model. Before model training, the preprocessed dataset needs to divide proportionally. Suppose you train the model with all the data. In that case, it may lead to training overfitting, so the data set is divided into a training set, validation set, and test set according to the ratio of 8:1:1. Training model with the training set then use the validation set to validate the model, adjust hyperparameters based on results, constantly adjust the model, find an optimal model by validation error. Select the current hyperparameters and model settings, add the data from the validation set to the training set to form a new training set, train a new model, and finally, use the test set to evaluate the model’s performance.

### 3.4 Correlation analysis

To train a robust and efficient predictive model, in addition to requiring a large amount of sample data, it is also necessary to select variables that impact changes in dust concentration. The selection of variables greatly affects the prediction accuracy of the model. So should analyze the correlation between dust concentration and other variables and choice appropriate variables. The paper uses the Pearson correlation coefficient to analyze the correlation between variables, calculated as follows:

$$r=\frac{\text{cov}(x,y)}{\sigma_{x}\sigma_{y}}=\frac{\sum_{i=1}^{n}\left(x_{i}-x^{*}\right)\left(y_{i}-y^{*}\right)}{\sqrt{\sum_{i=1}^{n}{\left(x_{i}-x^{*}\right)}^{2}\sum_{i=1}^{n}{\left(y_{i}-y^{*}\right)}^{2}}}$$ (11)

**Fig 5:**
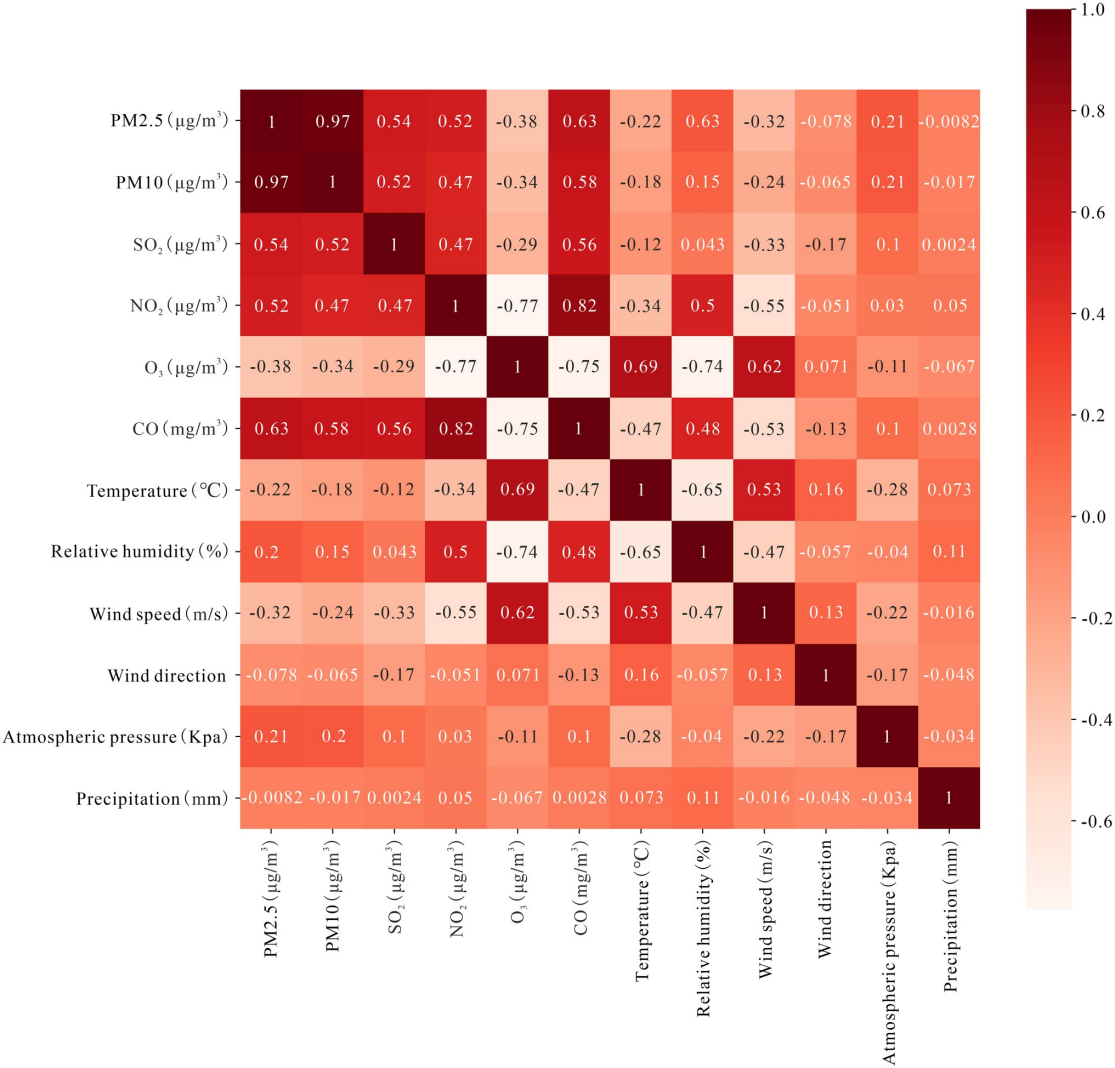
Pearson correlation analysis heat map of each variable.

In the formula, *x*_*i*_ and *y*_*i*_ are sample values, x* and y* are the sample mean, *and r* is a statistical indicator that reflects the correlation between variables. The value range of the correlation coefficient *r* is [–1,1]. When the absolute value of *r* is closer to 1, it indicates a stronger correlation between two variables. When r is 0, there is no correlation between the two variables. If *r*>*0*, there is a positive correlation between variables; if *r*<*0*, there is a negative correlation. We can draw a Pearson correlation analysis heat map of each variable to get Pearson correlation coefficients between variables. The Pearson correlation analysis heat map of each variable is Fig 5. Table 2 shows the results of correlation and significance analysis between PM2.5 and other variables.

**Table 2:** Correlation and significance analysis results between PM2.5 and other variables.

Variable	r	P	Variable	r	P
PM10	0.97**	< 0.01	Relative humidity	0.20**	< 0.01
SO_2_	0.54**	< 0.01	Wind speed	-0.32**	< 0.01
NO_2_	0.52**	< 0.01	Wind direction	-0.078*	< 0.05
CO	0.63**	< 0.01	Precipitation	-0.008	0.824
O_3_	-0.38**	< 0.01	Atmospheric pressure	0.21**	< 0.01
Temperature	-0.22**	< 0.01			

*. At the 0.05 level (two-tailed), significant correlation;

**. At the 0.01 level (two-tailed), significant correlation.

Analysis of the above table shows that, the correlation coefficient between PM2.5 and PM10 is 0.97, P<0.01, there is a significant positive correlation between the two, and the correlation is extremely high; the correlation coefficient between PM2.5 and SO_2_ is 0.54, P<0.01, there is a significant positive correlation between the two; the correlation coefficient between PM2.5 and NO_2_ is 0.52, P<0.01, there is a significant positive correlation between the two; the correlation coefficient between PM2.5 and CO is 0.63, P<0.01, there is a significant positive correlation between the two; the correlation coefficient between PM2.5 and O_3_ is -0.38, P<0.01, there is a significant negative correlation between the two; the correlation coefficient between PM2.5 and temperature is -0.22, P<0.01, there is a significant negative correlation between the two; the correlation coefficient between PM2.5 and relative humidity is 0.20, P<0.01, there is a significant positive correlation between the two; the correlation coefficient between PM2.5 and wind speed is -0.32, P<0.01, there is a significant negative correlation between the two; the correlation coefficient between PM2.5 and wind direction is -0.078, P<0.05, the two have a significant negative correlation, but the correlation is extremely low; the correlation coefficient between PM2.5 and precipitation is -0.008, the two are not significant and the correlation coefficient is close to 0, it is considered that there is no correlation between the two, this is related to precipitation intensity and precipitation duration, resulting in no apparent correlation with PM2.5; the correlation coefficient between PM2.5 and atmospheric pressure is 0.21, P<0.01, there is a significant positive correlation between the two.

Because the correlation between PM2.5 concentration and wind direction is extremely low, and there is no correlation with precipitation, so remove these two variables from the dataset. PM2.5 and PM10 are strongly correlated, and related research shows an inclusion relationship between PM2.5 and PM10. PM2.5 accounts for 50%~70% of PM10. Therefore, we use PM2.5 for the research on the prediction of road dust concentration in opencast coal mines.

## 4. Predictive model building

### 4.1 Data sample modeling

**Fig 6:**
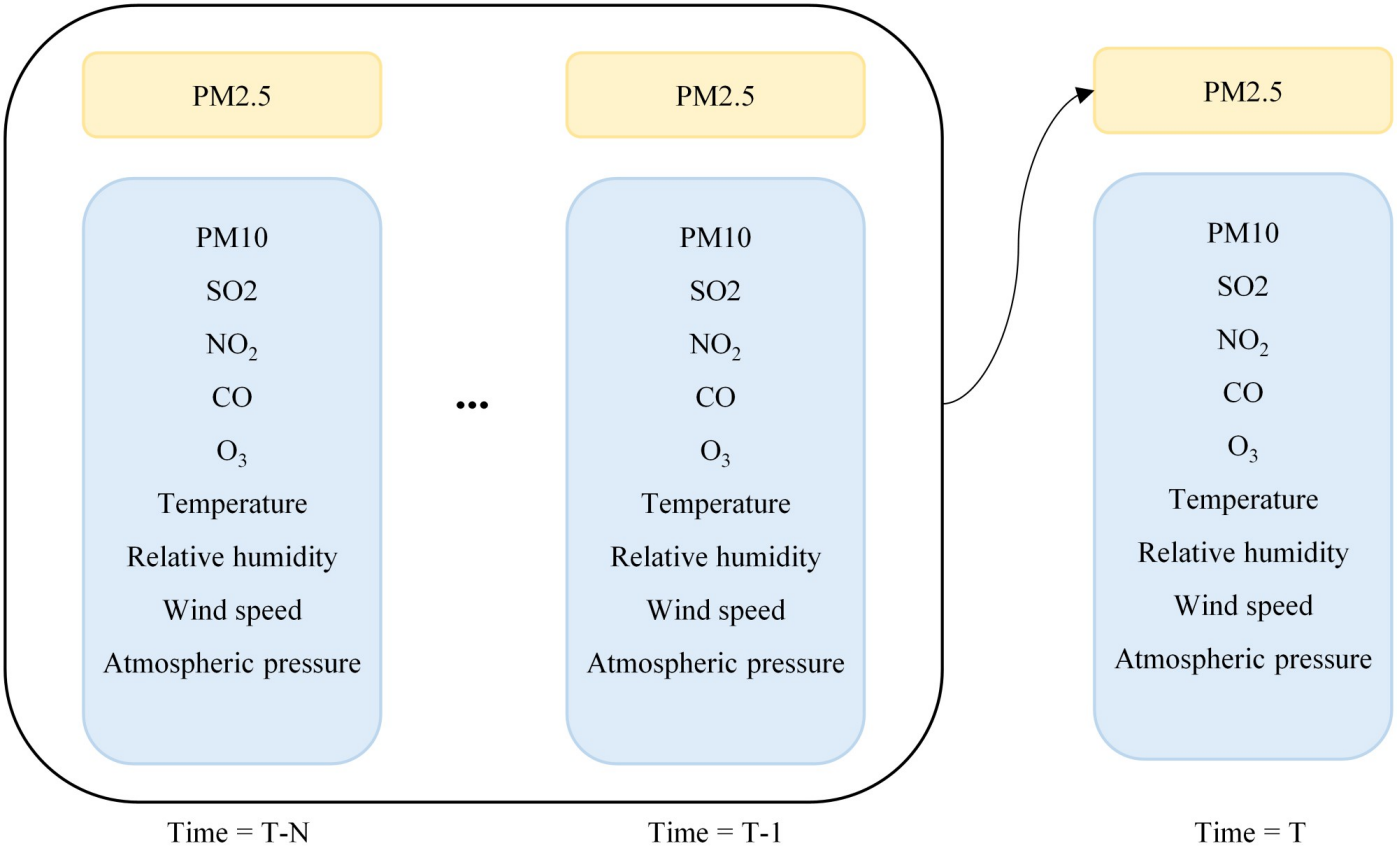
"Feature parameter-label" corresponding form.

The model trained in this paper is a supervised learning model that needs to train the data according to the ’ feature parameters—label ’ corresponding form to generate training samples. As shown in Fig 6, that is to use the data (feature parameters) at the historical time to predict the PM2.5 concentration (label) at the next time or the PM2.5 concentration multiple times after time T.

The data set in this paper is multivariate time series data, and the data before and after are strongly correlated. To preserve the correlation between adjacent data as much as possible, generate training samples by sliding time windows. The data at time T includes air quality data and meteorological data. The data at time T_i_ are T_i1_, T_i2_,…, T_ij_, j is the number of variables at each moment, set the first variable of each set of data as PM2.5 concentration. For example, T_i1_ represents the PM2.5 concentration at the time of T_i_.

**Fig 7:**
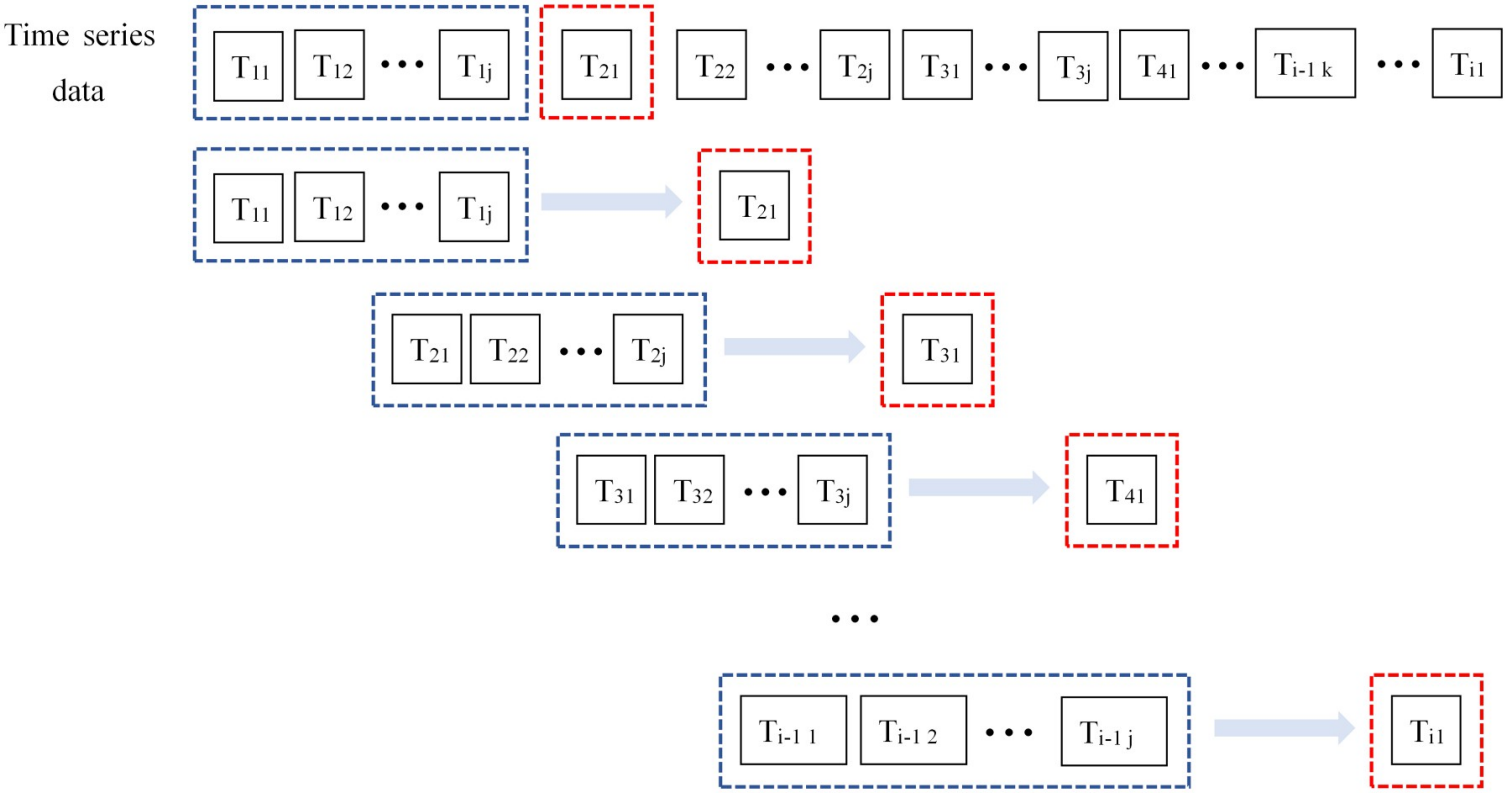
Multidimensional single-step prediction data sample generation process.

**Fig 8:**
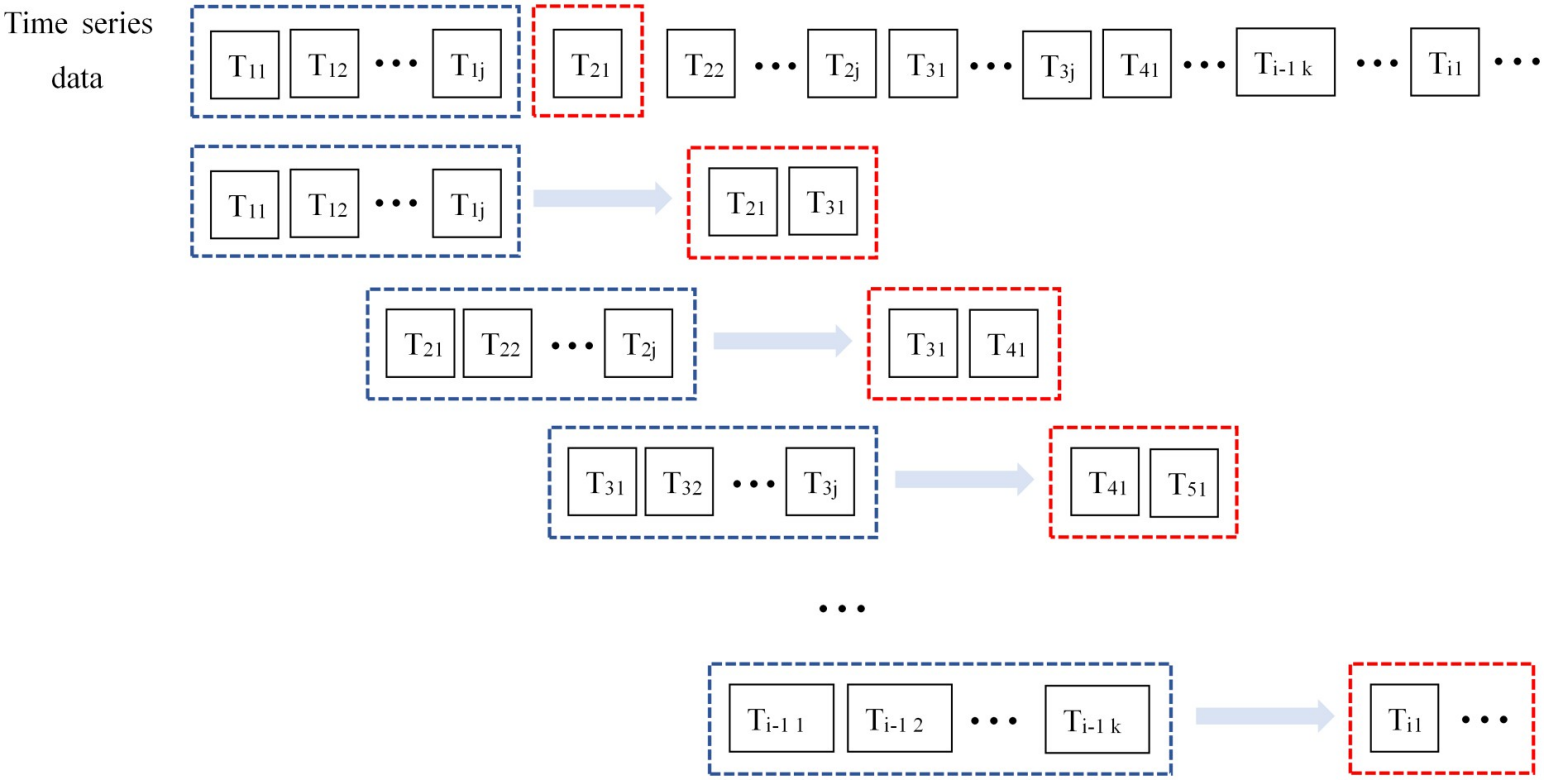
Multi-dimensional and multi-step forecasting data sample generation process.

(1) Assume that the characteristic parameters are all variable data at T_i_, labeled as PM2.5 concentration at T_i+1_ moment. This is a multidimensional single-step prediction. Fig 7 is the sample generation process of training data.

(2) Assume that the characteristic parameters are all variable data at time T_i_, labeled as PM2.5 concentration at T_i+1_ and T_i+2_. The sample generation process of training data is as follows. We can set the label as PM2.5 concentration at the time of T_i+1_, T_i+2_,…, T_i+n_. When the number of labels exceeds 1, it is a multi-dimensional multi-step prediction. Fig 8 is the sample generation process of the training data at this time.

### 4.2 Predictive model modeling

**Fig 9:**
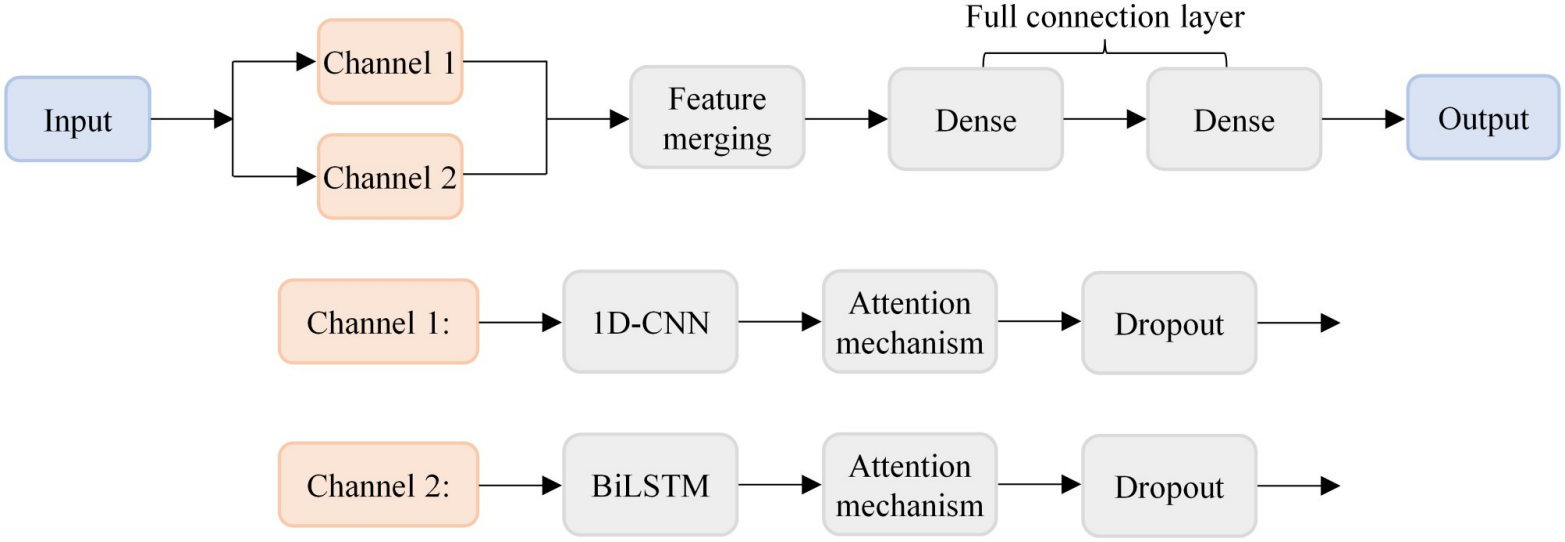
CNN-BiLSTM-attention parallel structure model.

The predictive model can build a hybrid model of the two structures. The first is a parallel structure, which divides the feature extraction process into two channels. Channel 1 uses 1D-CNN to locally extract nonlinear features of the data along the positive direction of the time axis. Channel 2 uses BiLSTM to extract the data’s bidirectional global temporal characteristics, assign weights to variables through an attention mechanism, and combine the two channels’ data features. Finally, the fully connected (Dense) lay can extract the correlation information between features, mapping to the output space for regression prediction and the output prediction result. Fig 9 shows the parallel structure model.

**Fig 10:**
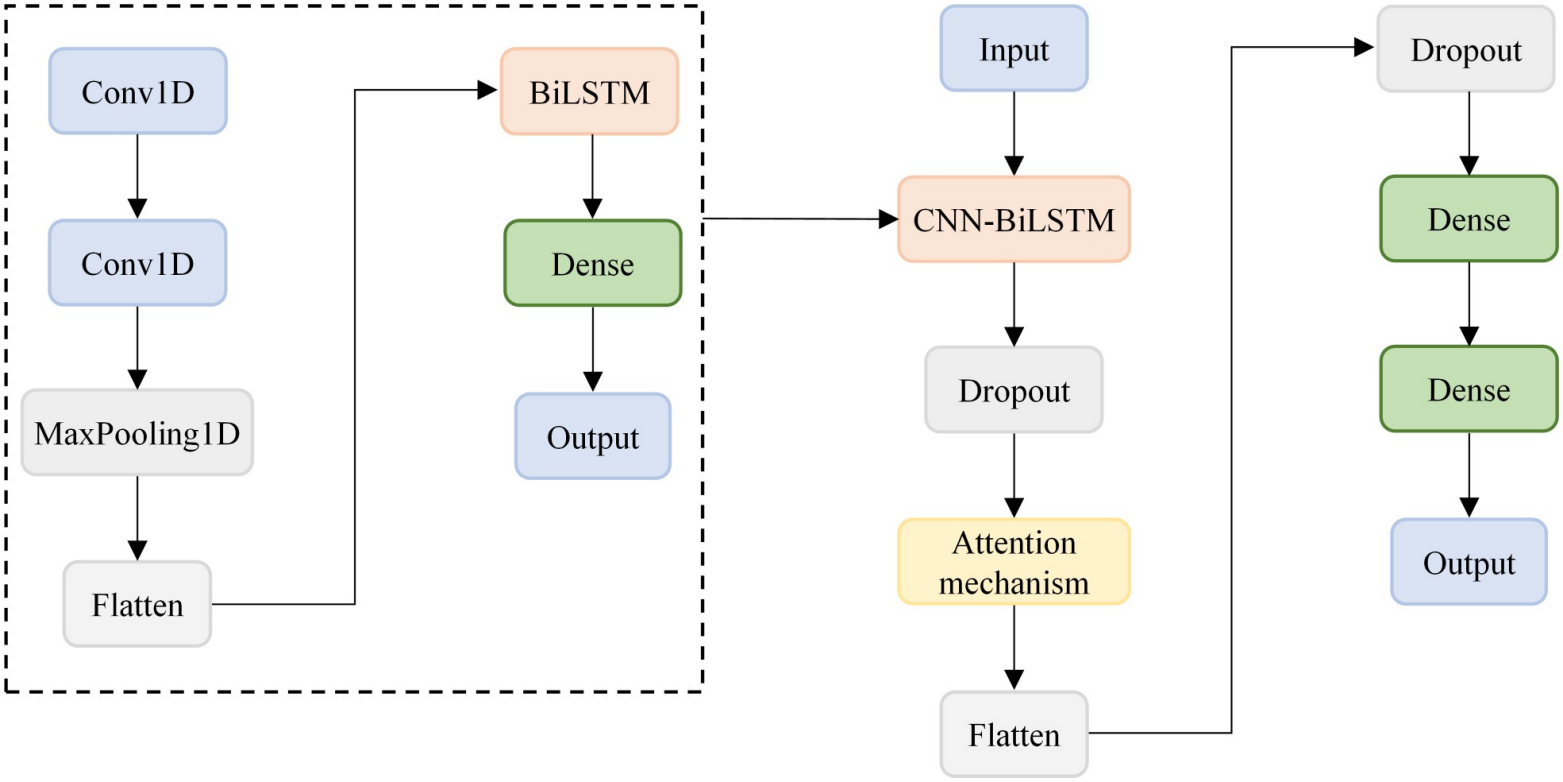
CNN-BiLSTM-attention serial structure model.

The second is the serial structure. The first part is the establishment of a hybrid model of CNN and BiLSTM. The second part is to introduce the attention mechanism. 1D-CNN and BiLSTM sequentially extract features in the same channel, superimpose data features to the same vector, then raise the attention mechanism and final output through the fully connected (Dense) layer. The next section of the article will set up a comparative experiment to compare the performance of the two structural mixture models and select the optimal structure of the model for further experiments. Fig 10 shows the CNN-BiLSTM-Attention serial structure model.

As shown in the figure above, set up a two-layer one-dimensional convolutional layer (Conv1D) in CNN to extract data features. The convolution kernel sizes are 32 and 64, respectively. The convolution kernel step size is 1. Compress and flatten the data through a one-dimensional max pooling layer (MaxPooling1D) and a Flatten layer. The data features extracted by CNN are input into BiLSTM to further mine the time series features in the data. Finally, the Dense layer completes the output of the CNN-BiLSTM part, this part of the output processes the input from the Dropout layer to the Attention layer, then goes through the Flatten layer, the Dropout layer. Two fully connected (Dense) layers for regression prediction complete the model’s training, and the Dropout layer prevents model overfitting. A dense layer can enhance data features. Deep Learning Model Based on Keras, we can use the Earlystopping mechanism to improve model training efficiency and prevent overfitting. At the same time, use the Glorot uniform initializer to initialize the neural network’s weights.

### 4.3 Evaluation indicators

To measure the prediction accuracy of the model, quantify the prediction effect of the model, select mean absolute error (MAE), root mean square error (RMSE), and coefficient of determination (R^2^) as the evaluation indicators of the prediction model, calculated as follows.


MAE=1n∑i=1nyi−yi^ (12)

RMSE=1n∑i=1nyi−yi^2 (13)

R2=1−∑i=1nyi−yi^2∑i=1nyi−y2 (14)

In the formula, n is the number of PM2.5 concentration data, *yi* is the actual value, *y^ i* is the predicted value, and *y* is the mean of the actual values. MAE and RMSE reflect the error between actual and predicted values. The smaller the value, the higher the prediction accuracy; R^2^ is the coefficient of determination, and the closer R2 is to 1, the better the model predicts.

## 5. Experiment

### 5.1 Performance comparison of different structural models

**Fig 11:**
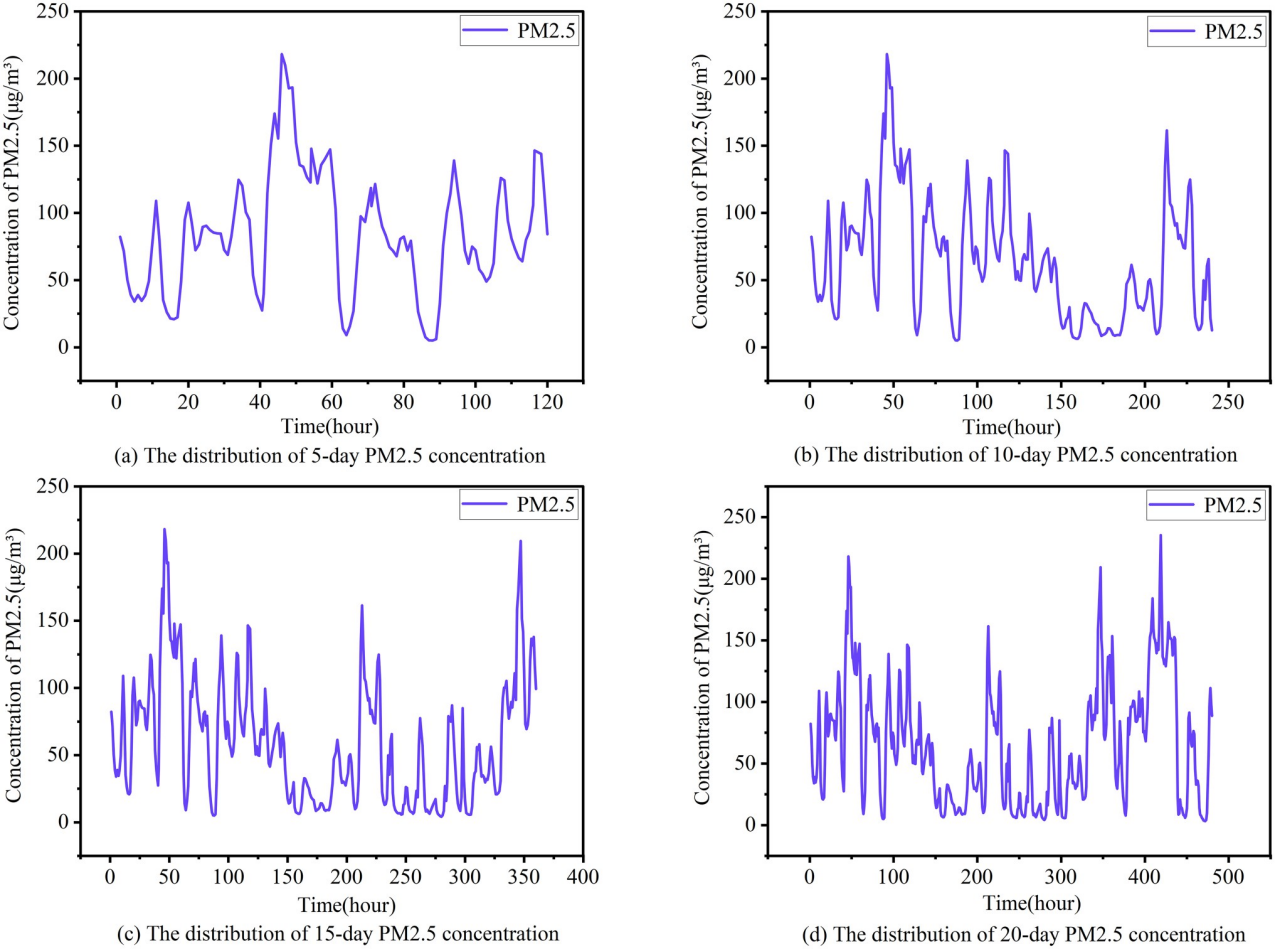
Changes in PM2.5 concentration.

For time series models, it is necessary to determine a suitable size of input and output variables to obtain higher prediction accuracy, how much historical data to input, and how much forecast data to output, a variable time span that is too long will increase the computational complexity of the model, the variable time span is too short. The model cannot fully extract the feature information between the data because the data of the data set has periodic changes. It can use periodicity as a characteristic parameter of a prediction model and find a suitable input and output variable size by determining the change period. We Plot changes in PM2.5 concentrations over 5, 10, 15, and 20 days in the first month of the dataset. In Fig 11, there is an obvious peak of PM2.5 concentration in 5 days, two obvious peaks of PM2.5 concentration in 10 days, three obvious peaks of PM2.5 concentration in 15 days, and four obvious peaks of PM2.5 concentration in 20 days, so determine five days as a cycle.

According to the determined period, set the size of different input and output variables and specific size settings are shown in Tables 3 and 4, and conduct many experiments on the prediction models of different structures. We divided the experiment into two groups. The experimental results are as follows.

1. Parallel structure model
2. Serial structure model

**Table 3:** Parallel structure model experimental results.

Input	Output							
	12h		24h		36h		48h	
	MAE	RMSE	MAE	RMSE	MAE	RMSE	MAE	RMSE
24*5h	5.917	6.505	6.149	6.565	6.297	6.449	6.485	6.892
24*10h	5.364	6.128	5.401	6.331	5.735	6.803	6.209	7.012
24*15h	5.210	5.895	3.677	4.126	4.118	5.033	5.143	6.249
24*20h	5.120	5.867	4.494	5.276	4.991	5.893	5.603	6.771

**Table 4:** Serial structure model experimental results.

Input	Output							
	12h		24h		36h		48h	
	MAE	RMSE	MAE	RMSE	MAE	RMSE	MAE	RMSE
24*5h	5.932	6.307	6.011	5.743	6.112	6.205	6.410	6.728
24*10h	5.141	5.836	5.219	6.013	5.467	5.811	6.034	6.475
24*15h	4.742	5.021	**2.694**	**3.384**	3.379	4.883	4.657	5.239
24*20h	4.776	5.466	3.895	4.026	4.891	5.664	5.548	6.337

Comparative analysis of experimental results, when the input and output are the same sizes, the prediction error of the serial structure model is lower than that of the parallel structure model. Better performance of the serial structure model when the input size is 24*15, and the output size is 24, the MAE and RMSE of the two models are the minima, and the prediction error is the lowest. Therefore, in the next experiment, select the CNN-BiLSTM-Attention serial structure model to continue the experiment while setting the input size to 24*15 and the output size to 24. The input matrix of the training model is 360*10 (24*15*10), 24 means 24 hours a day, 15 means 15 days of sample data input, 24 sets of sample data per day, each set of sample data contains ten variables, the output of the training model is 24h PM2.5 concentration. Table 5 is the hyperparameter setting of the model.

**Table 5:** Model hyperparameter settings.

Hyperparameter	Value
Filter size for CNN	12
Convolution Kernel size for CNN	32、64
Convolution kernel step	1
Padding	same
Activation function	ReLu
Neuron number for BiLSTM	20
Dropout rate for BiLSTM	0.2
Dropout rate for Flatten	0.5
Neuron number for Attention	360
Optimization function	Adam
Learing rate	0.001
Bacth size	200
Epoch	50

### 5.2 Short-term forecast

**Fig 12:**
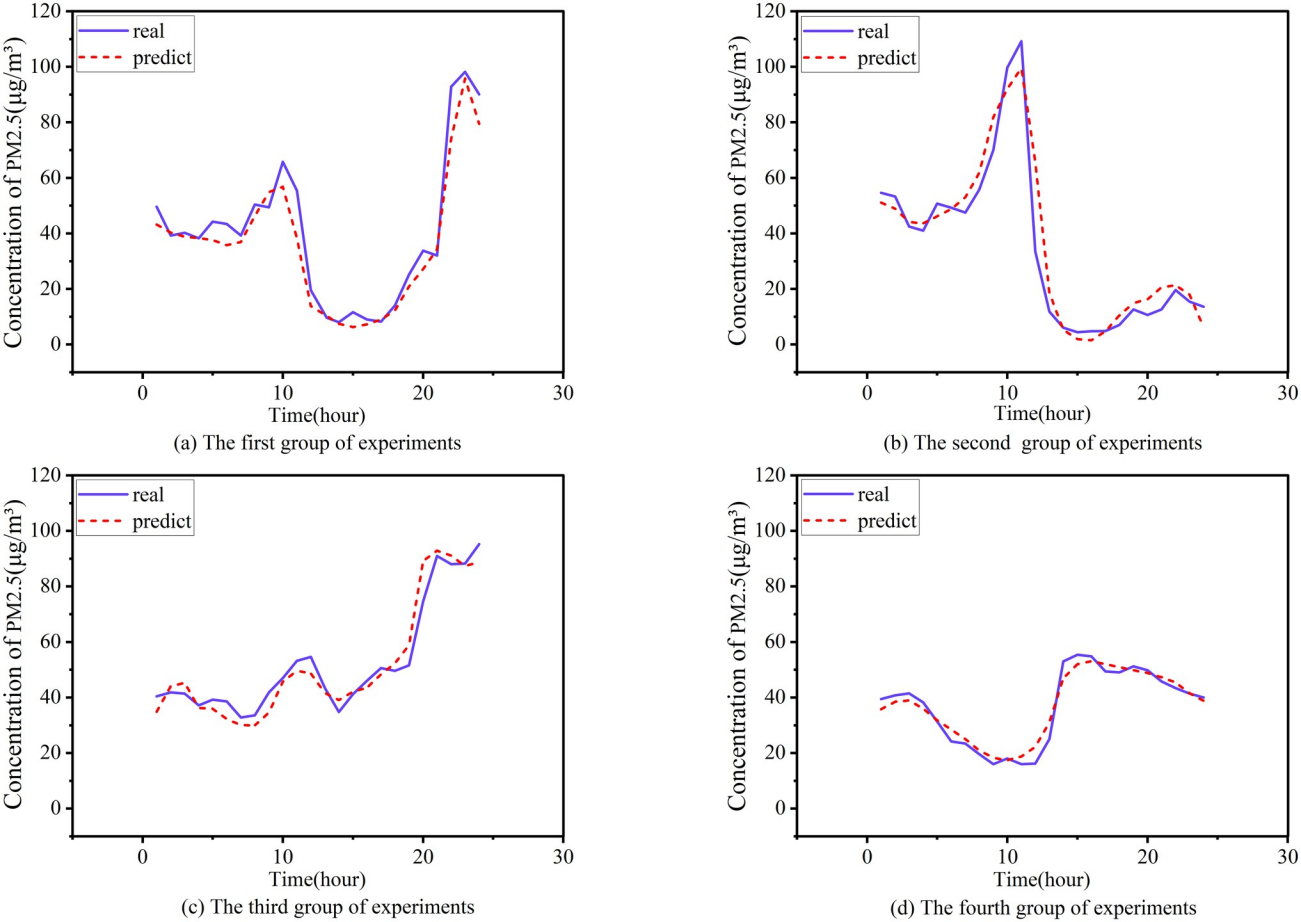
PM2.5 concentration prediction results.

To further verify the model’s performance, we randomly selected four groups of sample data from the test set for experiments. Each sample data group included 15-day time series data, and the PM2.5 concentration of 24 hours after 15 days was output. Fig 12 shows the comparison between the true and predicted PM2.5 concentrations. The solid blue line represents the actual value, and the red dotted line represents the predicted value. It can be seen intuitively from the figure that even in the case of large data fluctuations, the proposed model can still achieve better prediction results, and the model prediction value and the actual value are highly fitted. The evaluation indicators MAE, RMSE, and R2 of the four groups of experiments are 2.576, 3.068, and 0.964; 2.695, 3.237, and 0.952; 2.601, 3.113 and 0.967, 2.493, 2.994 and 0.969, and the average values of the four groups of evaluation indicators are 2.591, 3.103 and 0.963.

To verify whether the model has generalization, ten groups randomly selected from the test set for experiments were of sample data. We show the evaluation index results obtained in Table 6.

**Table 6:** Evaluation index results of 10 groups of experiments.

Serial number	MAE	RMSE	R^2^
1	3.157	4.032	0.946
2	3.056	3.948	0.950
3	2.654	3.457	0.957
4	4.369	5.231	0.941
5	5.034	6.124	0.935
6	2.327	2.983	0.963
7	2.489	3.112	0.961
8	1.736	2.035	0.967
9	1.301	1.901	0.972
10	0.819	1.013	0.981
**Average value**	2.694	3.384	0.957

The evaluation index results in the above table show that, except for the large prediction error results of individual experiments, the overall experiments have achieved good prediction results. To verify the superiority of the model proposed in this paper, we selected traditional regression, machine learning, single deep learning, and hybrid deep learning models for experiments with the same dataset. Namely Lasso regression, SVR, XGBoost, LSTM, BiLSTM, CNN-LSTM, CNN-BiLSTM, and CNN-BiLSTM-Attention model, adjust the hyperparameters of all models to the optimal for comparative experiments. Figs 5.3 and 5.4 compare the actual and predicted values for different models.

**Fig 13:**
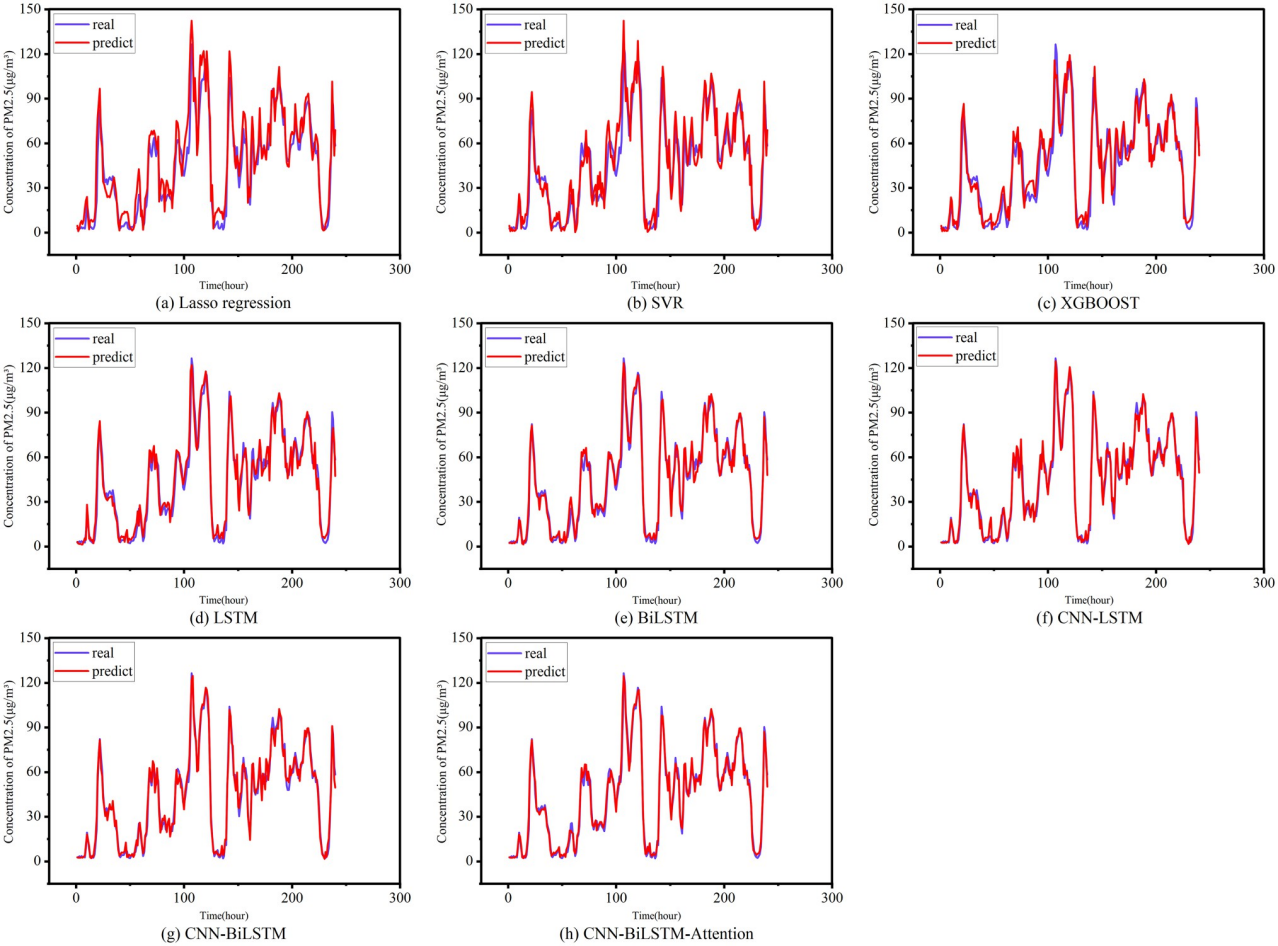
Comparison of the true and predicted values of each model.

**Fig 14:**
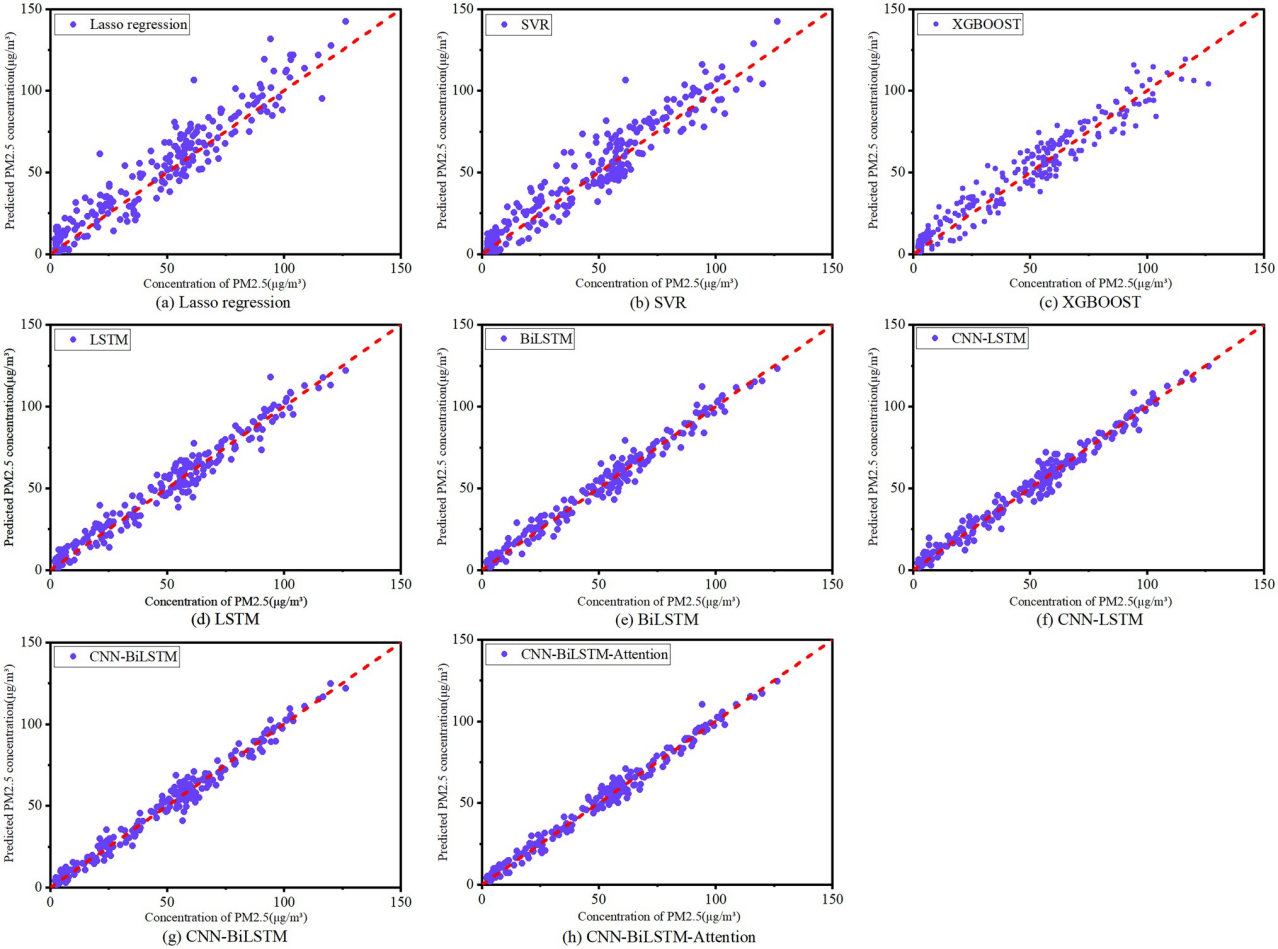
True and predicted values of different models.

Fig 13 is a line chart comparing each model’s actual and predicted values of PM2.5 concentrations. When the true value of PM2.5 concentration is between 15 and 105 μg/m3, the prediction effect of each model is better, and the fitting degree between the actual value and the predicted value is high. When the actual value of PM2.5 concentration is less than 15μg/m3 and greater than 105μg/m3, the fitting effect of the model is poor. The result shows that the prediction effect of the traditional model Lasso regression is the worst, and the curve change of the predicted value has a large deviation from the actual value. Fig 14 is the scatter plot of the actual and predicted values of PM2.5 concentration in each model. When the scatter is closer to the diagonal line, the error between the actual and predicted values is smaller. It can be seen intuitively from the figure that the prediction effect of the traditional model is poor, followed by the machine learning model. Still, the prediction effect of SVR is worse than that of XGBoost, and the prediction effect of the deep learning model is significantly better than that of the traditional and machine learning models. The prediction accuracy of the hybrid deep learning model is better than that of the single deep learning model. The scatter of CNN-BiLSTM-Attention is closer to the diagonal than CNN-LSTM and CNN-BiLSTM, indicating that its prediction accuracy is higher.

**Fig 15:**
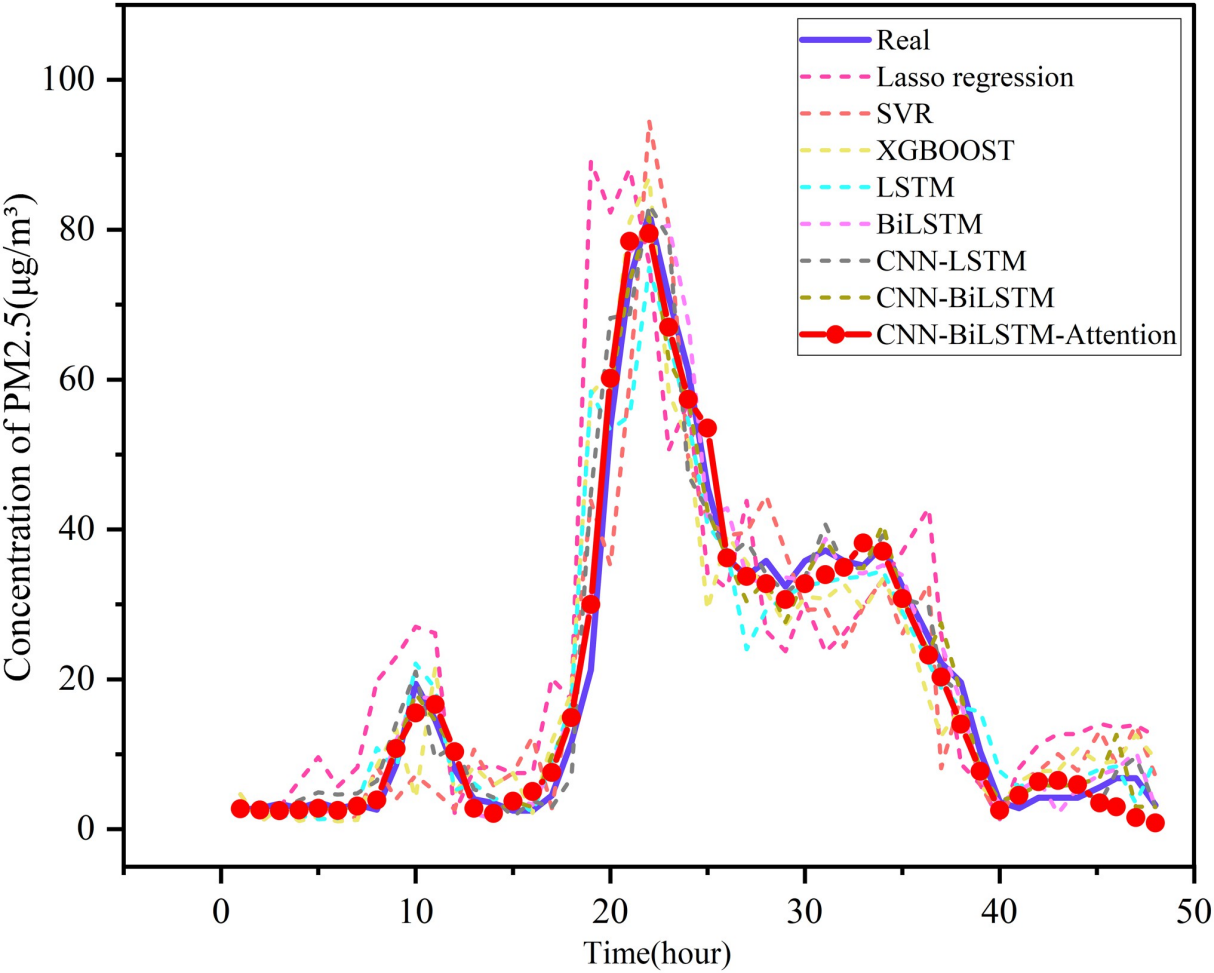
Comparison of the true and predicted values of each model.

To see the comparison between the prediction effects of each model more intuitively, select the real values and predicted values of the first 48 groups of each model and draw their line graphs. As shown in Fig 15, blue represents the actual value, and the red dotted line represents the predicted value of the CNN-BiLSTM-Attention hybrid model. The predicted value obtained by the model proposed in this paper is the closest to the actual value, and the prediction accuracy is the highest. The model obtains good prediction results because it can extract nonlinear features locally and two-way global time features of data and assign weights to variables through the attention mechanism to mine data information fully.

### 5.3 Long-term forecast

**Fig 16:**
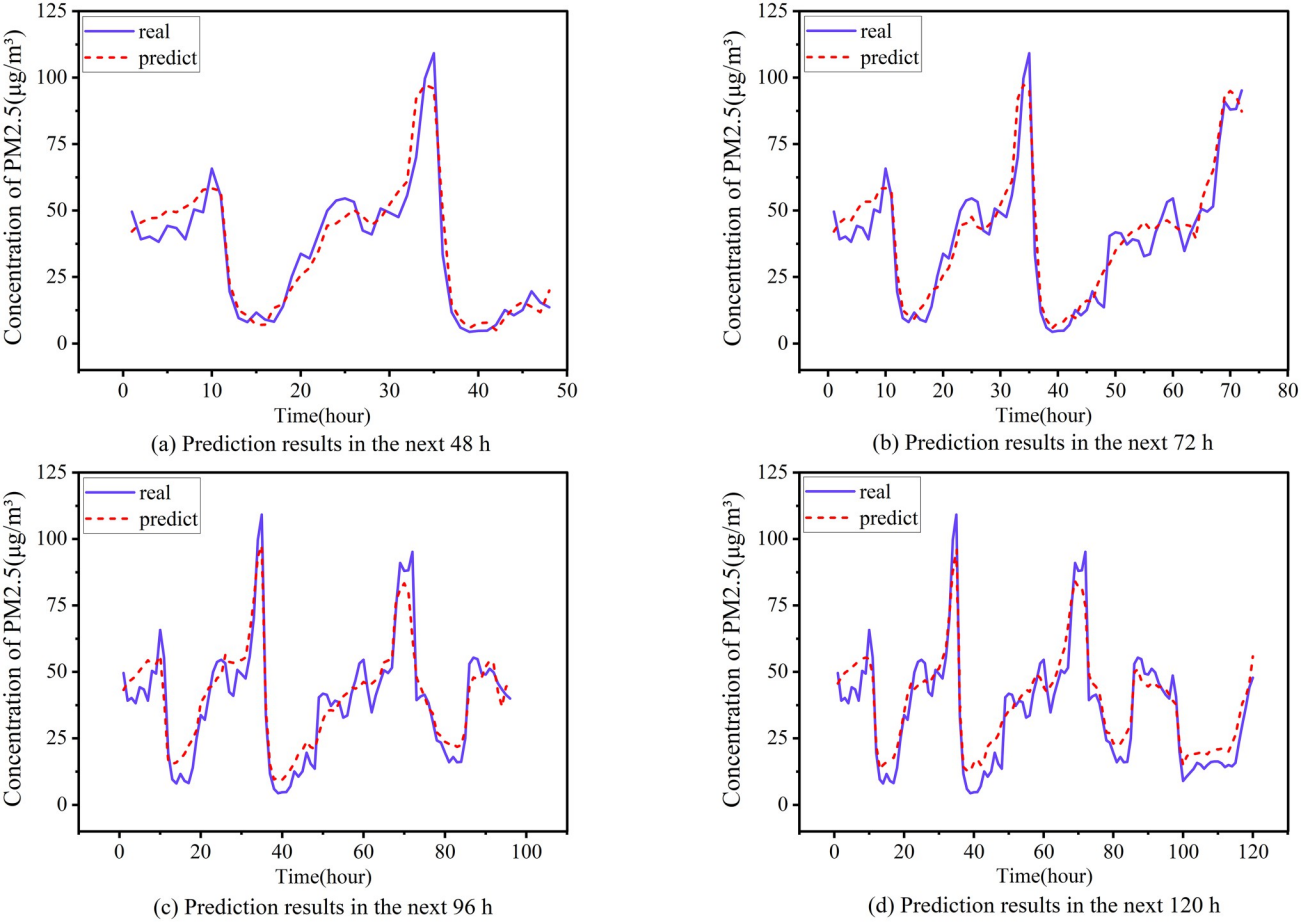
Long-term prediction results of PM2.5 concentration.

To test the robustness of the proposed model, experiments for long-term prediction through the model are also required. This section sets prediction experiments of 48h, 72h, 96h, and 120h. As seen from Fig 16, as the prediction time increases, the prediction error also increases gradually, but the prediction result can still fit the data trend. When the prediction time gradually increases, the correlation between the predicted data (output) and the known data (input) decreases, the influence of each variable on PM2.5 concentration will gradually decrease, and the weight assigned to the variable by the attention mechanism will also decrease. This results in a decrease in prediction accuracy and an increase in prediction error. At the same time, for all the comparison models, we performed the same long-term prediction experiments, and we compared the performance of the models through the evaluation indicators MAE and RMSE. Table 7 shows the evaluation indicators of each model under different prediction output step sizes. The table shows that with the increase in prediction time, the prediction errors of each model gradually increase, and the results of the hybrid model proposed in this paper are still optimal at different prediction times. Therefore, this model is also suitable for long-term prediction.

**Table 7:** Long-term prediction and evaluation indicators of each model.

Model	Output							
	48h		72h		96h		120h	
	MAE	RMSE	MAE	RMSE	MAE	RMSE	MAE	RMSE
Lasso regression	10.975	11.973	11.730	13.267	12.959	14.426	14.012	16.581
SVR	10.582	11.478	11.139	12.638	11.678	13.851	12.689	15.784
XGBOOST	9.033	12.017	10.867	13.980	12.835	16.375	13.499	16.823
LSTM	7.934	10.113	9.101	12.037	10.549	12.938	12.893	15.318
BiLSTM	7.129	9.356	8.226	10.519	9.660	12.103	12.130	14.694
CNN-LSTM	6.438	8.376	7.859	9.661	9.126	10.467	11.871	13.251
CNN-BiLSTM	5.823	7.931	6.742	8.519	6.921	8.919	7.532	10.476
CNN-BiLSTM-Attention	4.657	5.239	6.035	7.254	6.348	8.658	6.957	8.985

### 5.4 Measured data verification

**Fig 17:**
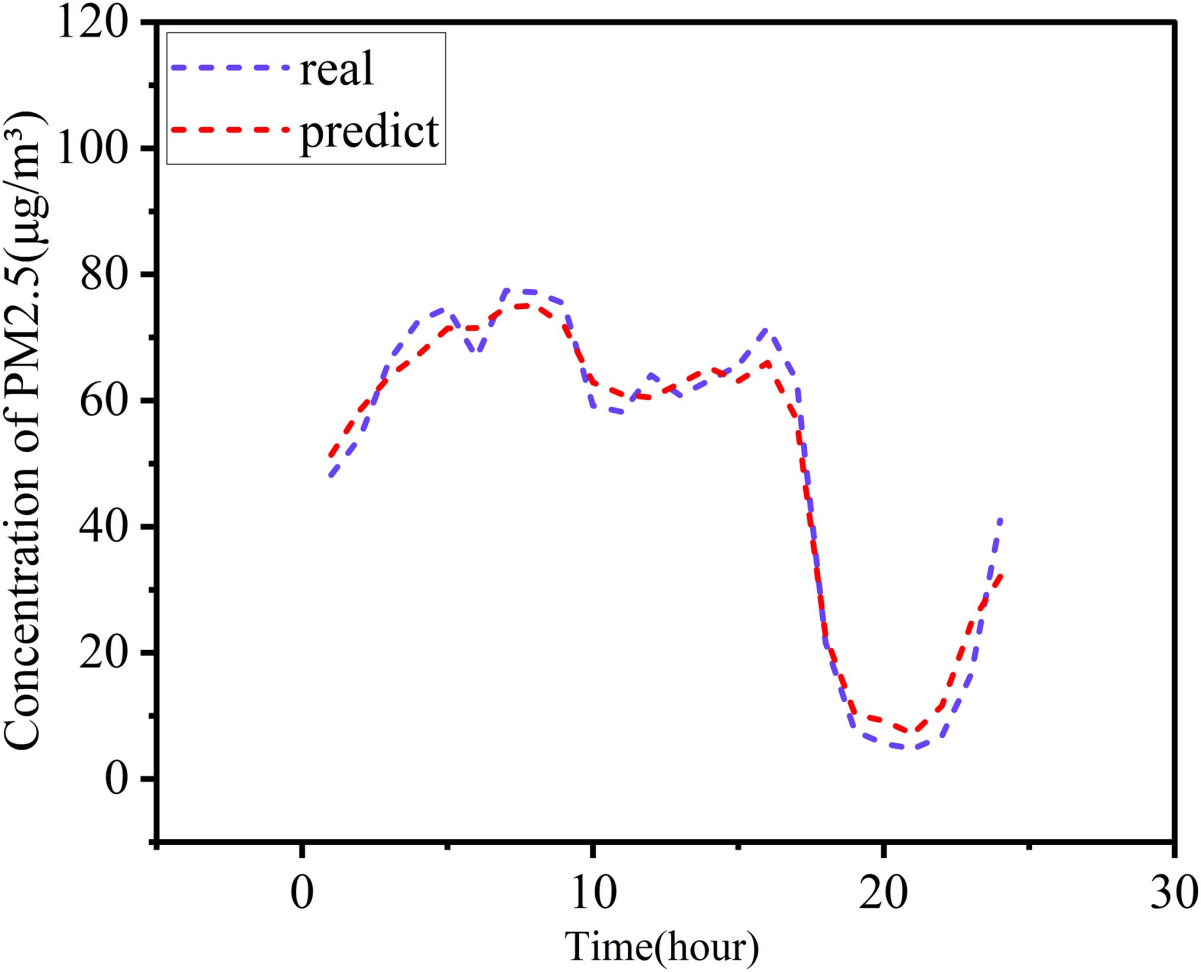
Comparison of measured data and predicted value.

After the model training and testing are completed, the relevant data show that the model performs well. We use field-measured data to verify the model’s performance in practical applications. We set the monitoring point on the side of the transportation road of the tailings reservoir in the open-pit coal mine. The monitoring point is about 5m away from the center of the road, and the monitoring height is about 1.5m. The data obtained by monitoring predicts the PM2.5 concentration in the next 24 hours. Fig 17 compares the actual and predicted values. The figure shows that the model predicted value and the actual value have a high degree of fit, and the evaluation indicators MAE, RMSE, and R2 are 3.127, 3.989, and 0.951.

## 6. Conclusion

Based on the hourly air quality and meteorological data of an open-pit coal mine in Tongliao City, Inner Mongolia Autonomous Region, from January 1, 2020, to December 31, 2021, this paper analyzes the correlation between each variable and dust concentration. We screened the appropriate variables for the prediction model, and a multivariate mixed dust concentration prediction model was established using the deep learning method and verified by field-measured data. This paper draws the following conclusions:

(1) The variable correlation analysis shows that PM2.5 and PM10 have a strong correlation. Therefore, PM2.5 is the primary method to establish a prediction model of road dust concentration in an open-pit coal mine. At the same time, the correlation analysis results show that the correlation between pollutant variables (PM10, SO_2_, NO_2_, CO, O_3_) and PM2.5 concentration is higher than that between meteorological variables (temperature, relative humidity, wind speed, atmospheric pressure) and PM2.5 concentration. This result also shows that it is necessary to strengthen the prevention and control of other pollutants in the air in the process of road dust control in open pit mines.

(2) This paper establishes a CNN-BiLSTM-Attention prediction model with parallel and serial structures. Through many experiments, the results show that the performance of the serial structure model is better than that of the parallel structure model. When the input size is 24*15, and the output size is 24, the model has the best prediction performance and the lowest prediction error. The evaluation indicators MAE, RMSE, and R^2^ are 2.694, 3.384, and 0.957. We compare the model established in this paper with the traditional regression, machine learning, and single deep learning models for prediction experiments with different time steps (48h,72h, 96h, and 120h). The experimental results show that the prediction performance of the CNN-BiLSTM-Attention model is optimal at each time step, and the hybrid model has good generalization and robustness.

(3) As verified by the measured data, the prediction results are good. The results indicate that we can use the model for the on-site prediction of dust concentration. Evaluation index MAE, RMSE, and R^2^ are 3.127, 3.989, and 0.951.
